# Sex-associated circadian signaling signatures in the lateral hypothalamus

**DOI:** 10.3389/frsle.2026.1895831

**Published:** 2026-09-16

**Authors:** Sara K. Pintwala, Patrick M. Fuller

**Affiliations:** Department of Neurological Surgery, Davis School of Medicine, University of California, Davis, Davis, CA, United States

**Keywords:** gene expression, lateral hypothalamus (LH), melanin-concentrating hormone (MCH) neurons, orexin neurons, sex differences, sex dimorphism, single nucleus RNA sequencing

## Abstract

**Introduction:**

The lateral hypothalamus (LH) is a central integrative hub coordinating arousal, feeding, metabolism, stress responsiveness, and sleep-wake state transitions. These functions are not static across the 24-hour cycle, but instead are dynamically gated according to circadian phase and internal physiological state. Although sex differences have been documented across many homeostatic and behavioral domains linked to LH function, the molecular substrates underlying these differences within defined LH neuronal populations remain poorly understood.

**Methods:**

Here, using single-nucleus RNA sequencing, we examined the expression patterns of core clock genes and circadian signaling-associated pathways across transcriptionally resolved populations of female- and male-derived LH neurons.

**Results:**

While the overall cellular architecture of the LH was highly conserved between sexes, we identified distributed and cell-type-specific differences in the expression of genes associated with intrinsic circadian regulation and sensitivity to canonical circadian neuromodulatory pathways, including vasoactive intestinal peptide, arginine vasopressin, neuromedin signaling, and melatonin receptors. These differences were observed across glutamatergic, GABAergic, orexinergic, and melanin-concentrating hormone-associated neuronal populations, suggesting that female and male LH circuits may differentially integrate temporal and physiological information despite preserving shared cellular organization.

**Discussion:**

Rather than indicating sex-specific neuron classes, our findings support a model in which subtle but coordinated shifts in molecular signaling architectures are linked to female- and male-derived LH neurons. These data provide a framework for understanding how temporal regulation of competing homeostatic drives may differ between sexes and underscore the importance of incorporating sex as a biological variable in transcriptomic analyses of hypothalamic circuits.

## Graphical Abstract

**Figure:**
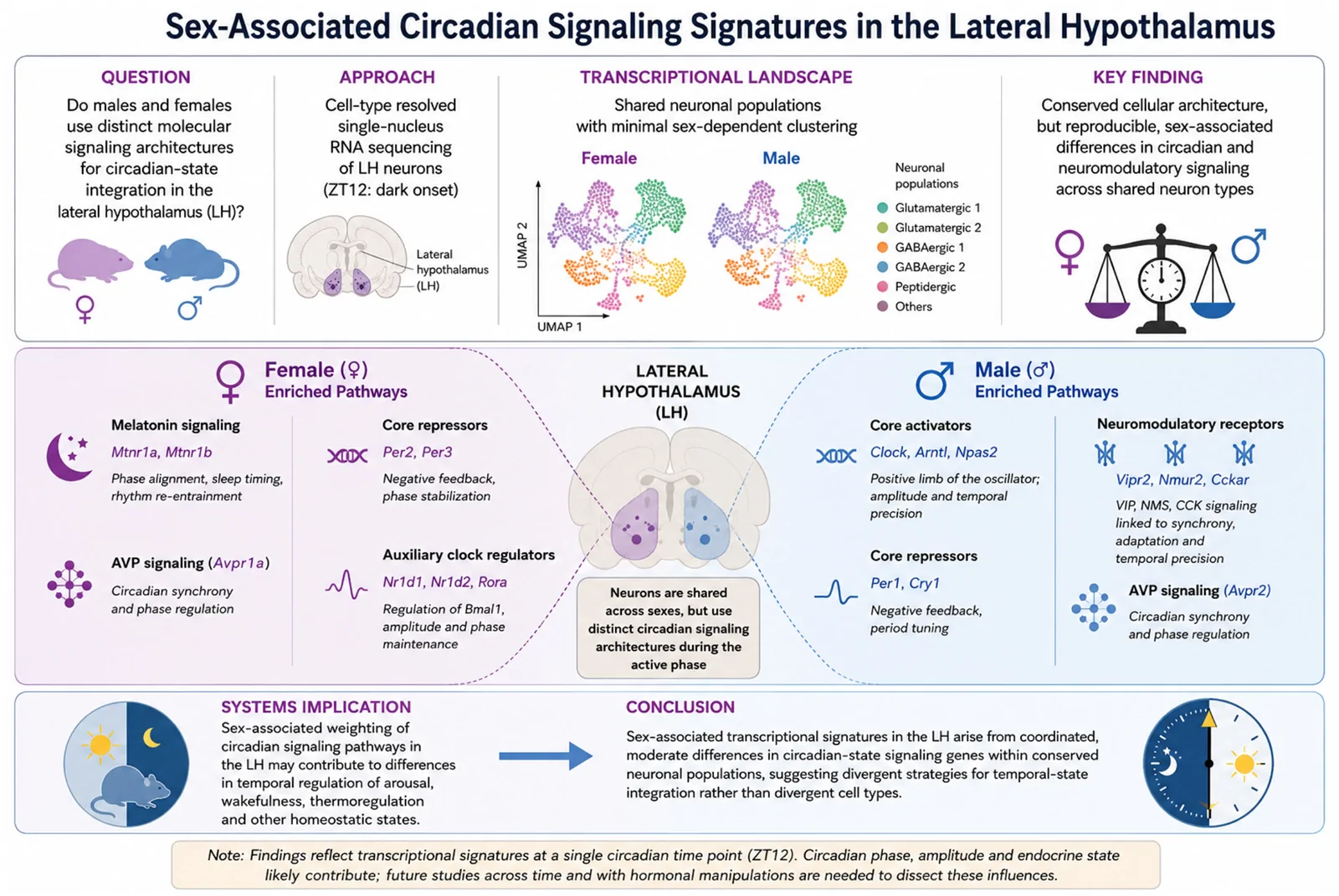
Created in BioRender. Fuller, P. (2026). https://BioRender.com/kknbbjf.

## Introduction

In mammals, circadian rhythms are generated by a hierarchical timing system tuned to the master pacemaker in the suprachiasmatic nucleus (SCN) which integrates external information to synchronize molecular clocks throughout the central nervous system (CNS) and periphery (Moore and Eichler, 1972; Stephan and Zucker, 1972). Through neural and humoral outputs the SCN aligns physiology and behavior with the external light-dark cycle, tuning these processes to an approximately 24 h period. To exert the effects of circadian timing for diurnal processes, the SCN forms a distributed circadian regulatory network in the CNS through secondary oscillators, including hypothalamic nuclei like the dorsomedial hypothalamus (DMH). This regions integrates SCN-derived information regarding time of day by way of the subparaventricular zone (SPZ) to control circadian-linked behaviors (Mieda et al., 2006) and acts as an integrative node to coordinate circadian outputs (Tahara et al., 2010). Recent evidence suggests that the DMH contains molecularly-defined populations that regulate rhythmic arousal in a clock-dependent manner (Liu et al., 2023), supporting a model where the SCN translates circadian information to auxiliary nodes for network-level regulation of rhythmic processes. While the SCN and auxiliary oscillators like the DMH are recognized as key regulators of circadian physiology and behavior, considerably less is known about the contribution of other downstream circuits within the circadian network which similarly control diurnal processes.

The lateral hypothalamus (LH) occupies a central position within the neural architecture controlling arousal, feeding, metabolism, thermoregulation, motivated behaviors, stress responsiveness, and sleep-wake state transitions (Astafev et al., 2024; Aton et al., 2005). Rather than functioning as a dedicated center for any single behavior, the LH is increasingly recognized as an integrative hub that coordinates competing homeostatic drives according to both internal physiological demands and external environmental conditions. Through extensive reciprocal connectivity with hypothalamic, limbic, brainstem, and cortical structures, including the SCN (Hung et al., 2025) and DMH (Chou et al., 2003), LH neurons participate in the selection, maintenance, and suppression of behavioral and autonomic states essential for survival (Cheon et al., 2025; Yamashita and Yamanaka, 2017; Herrera et al., 2017). Importantly, these functions unfold within a temporally structured physiological landscape. Across the 24-h cycle, the probability of wakefulness, feeding, exploration, thermoregulation, and defensive responding fluctuate according to circadian phase (Van Drunen and Eckel-Mahan, 2021). In addition to intrinsic clock-associated transcriptional programs (Abe et al., 2002), LH neurons express receptors for several neuromodulatory pathways associated with circadian timing and state regulation, including vasoactive intestinal peptide (VIP) (Ishihara et al., 1992), arginine vasopressin (AVP) (Ostrowski et al., 1994), gastrin-releasing peptide (GRP) (Kamichi et al., 2005), melatonin-associated receptors (Sharma et al., 2018) and related modulators implicated in rhythmic physiological coordination (Cheon et al., 2025; Cho et al., 2012). Together, these systems provide a potential substrate through which the LH may influence the prioritization of circadian-linked processes by gating competing homeostatic demands. Because of this, the LH is well-positioned to integrate temporally organized signals with information regarding physiological state through interactions with canonical circadian regulatory systems, including pathways and functions linked to the SCN.

Because the LH integrates neural, humoral, and environmental signals, other sources of physiological variation should be considered when assessing LH function. Of these competing demands, sex differences have been documented across many physiological and behavioral domains linked to LH function, including stress responsivity (Depaauw-Holt et al., 2025), motivated behaviors (Dawson et al., 2023) sleep, wakefulness, metabolic regulation, and circadian organization (Bailey and Silver, 2014; Begemann et al., 2025; Lok et al., 2024). Despite these observations, sex dimorphism in the LH is highly understudied even though the critical role of this region in controlling these modalities is widely recognized (Astafev et al., 2024; Aton et al., 2005). Sex dimorphism is documented in SCN across several domains, including sex-dependent synaptic organization (Güldner, 1982, 1984) brain volume (Robinson et al., 1986), distribution in the preoptic area (Hofman et al., 1988), neurochemical composition (Hofman et al., 1996), and neuromodulatory oscillations (Mahoney et al., 2009). Sex hormone receptors in the SCN exhibit cell type specificity and regional localizations (Jahan et al., 2025; Karatsoreos et al., 2007; Butler et al., 2012; Vida et al., 2008) and are associated with sex-specific signaling motifs for firing synchrony (Schlaeger et al., 2024) and circadian phase shifts (Karatsoreos et al., 2007). These dimorphisms are yet fully understood, but may underlie observed sex differences (Anderson and FitzGerald, 2020) in circadian period (Duffy et al., 2011), amplitude (Santhi et al., 2016), and rhythmic gene expression (Anderson and FitzGerald, 2020; Talamanca et al., 2023; Astafev et al., 2024). Although the overall anatomical organization of the LH is largely conserved between the sexes (Mickelsen et al., 2019) emerging evidence indicates that LH function is sex-specific, for the control of reproductive and social behaviors (Kato et al., 2021), and dynamically modulated by sex hormones, including estrogen signaling in the LH to shape motivated (Contesse et al., 2025) and aversive behaviors (Calvigioni et al., 2023). Despite accumulating evidence for the influence of sex on circadian-linked behaviors and physiological processes, comparatively little is known about the cellular and molecular substrates underlying these differences. An important unresolved question is whether sexual dimorphism within the LH reflects fundamentally distinct neuronal populations or instead emerges through distributed modulation of shared circuit architectures.

Recent advances in single-cell and single-nucleus transcriptomics have enabled increasingly refined characterization of hypothalamic cell populations and their molecular diversity (Mickelsen et al., 2019, 2017; Chen et al., 2017; Wu et al., 2025; Todd et al., 2020). These approaches have revealed that neuronal identity is shaped not only by neurotransmitter phenotype and developmental lineage, but also by differential expression of molecular features that regulate physiological state, neuromodulatory sensitivity, and temporal dynamics (Shainer et al., 2025; Pintwala et al., 1972; Scala et al., 2020). In this context, transcriptomic analyses provide an opportunity to examine whether molecular components associated with circadian integration and intrinsic clock regulation exhibit sex-dependent organization across transcriptionally resolved LH neuron populations.

Here, using single-nucleus RNA sequencing (snRNAseq) in female- and male-derived LH neurons, we examined the expression of core clock genes and circadian signaling-associated pathways across glutamatergic, GABAergic, orexinergic, and melanin-concentrating hormone (MCH)-associated neuronal populations. We hypothesize that female- and male-derived LH neurons exhibit distinct transcriptional signatures within core clock genes and circadian neuromodulator associated signaling pathways, which may reflect biological sex, endocrine state, circadian phase, or interactions among these variables. Although the overall cellular architecture of the LH was highly conserved between sexes, we identified distributed and cell-type-specific differences in genes associated with intrinsic circadian regulation and sensitivity to canonical circadian neuromodulatory pathways. Rather than supporting the existence of sex-specific LH neuron classes, our findings suggest that sexual dimorphism may emerge through subtle but coordinated shifts in molecular signaling architectures linked to temporal-state integration. These data provide a framework for understanding how circadian and homeostatic information may be differentially weighted across sexes within a major hypothalamic integrative hub.

## Results

### Single-nucleus RNA sequencing in the LH

To profile gene expression in the lateral hypothalamus (LH) of female (*n* = 4) and male (*n* = 4) mice, we used snRNA-seq. Tissue was extracted at dark phase onset (zeitgeber time, ZT 12) between 19:00 and 19:40 (Figure 1a), a biologically relevant transition period, alternating between female and male mice. Brains were sectioned between −1.0mm and −2.0mm from bregma and the LH was bilaterally extracted using a 1.0 mm tissue biopsy punch. Female- and male-derived nuclei were submitted to 10x Genomics for snRNA-seq using the Chromium GEM-X Single Cell 3′ v4 droplet-based gene expression assay on the AVITI platform. This resulted in 47,909 female-derived LH nuclei and 37,191 male-derived LH nuclei.

**Figure 1:**
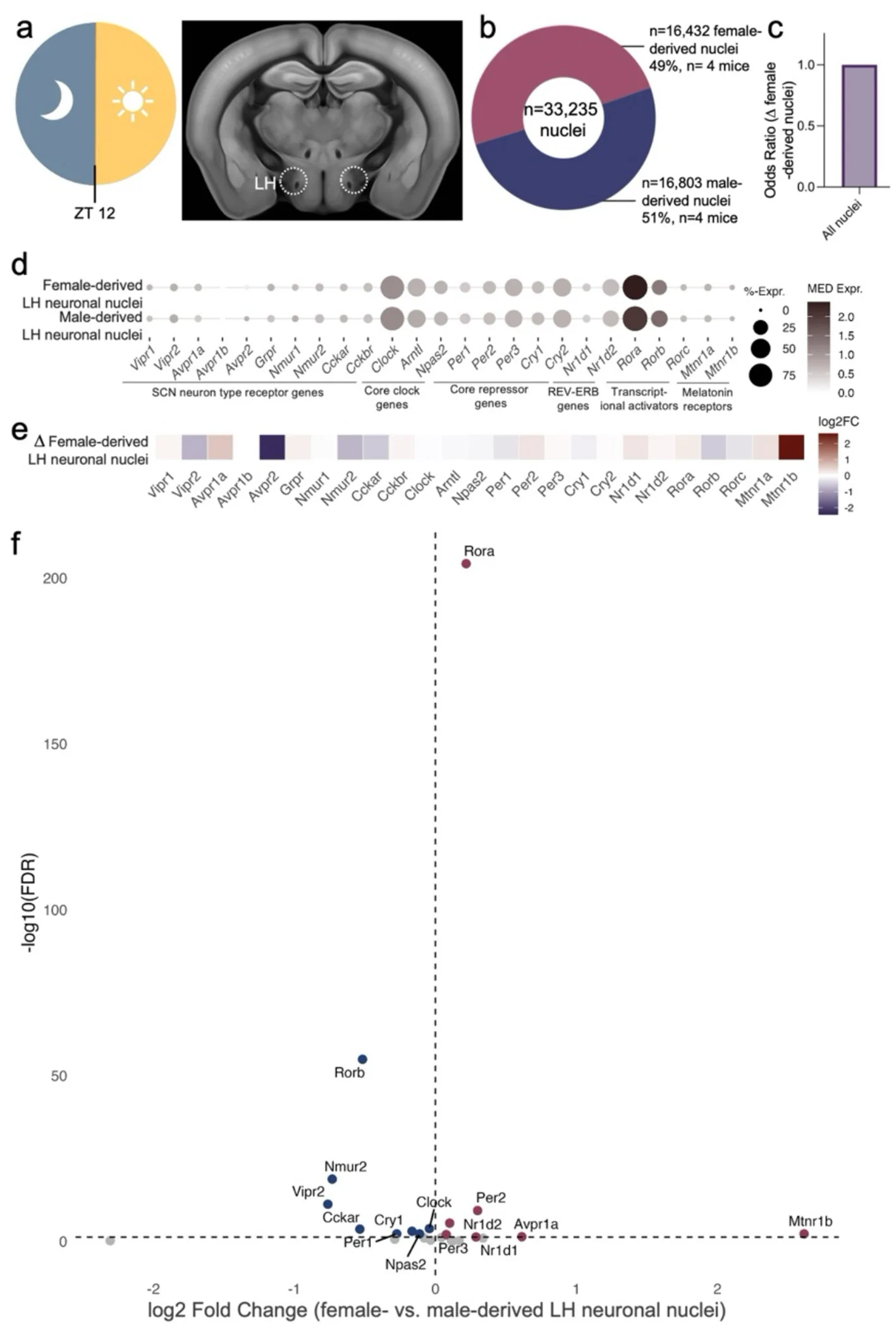
Comparing clock and circadian signaling-associated gene expression across the sexes in LH neurons. **(a)** At dark phase onset (ZT 12) the LH was bilaterally dissected from mice alternating between the sexes. Representative image of the LH is visualized using the Allen Mouse Brain Common Coordinate Framework version 3 (CCFv3; Wang et al., 2020). Samples were processed according to sex (*n*= 4 mice per group) for snRNAseq. **(b)** A total of 33,235 nuclei were studied, 16,432 nuclei derived of female mice and 16,803 nuclei derived of males. **(c)** Odds ratio comparing female- and male-derived nuclei counts according to sex. **(d)** Dot plot with median normalized expression levels (MED Expr.) and prevalence (percent expression, %-Expr.) of clock and circadian signaling-associated genes of interest (GOIs) in both sexes. **(e)** Heatmap depicting GOI expression as log2 Fold Change (log2FC) for effect sizes. **(f)** Volcano plot of differentially expressed GOIs (Wilcoxon rank-sum test; false discovery rate, FDR; Benjamini–Hochberg correction, *p*-value < 0.05).

From the resulting dataset, ambient RNA was removed with SoupX (Young and Behjati, 2020) and doublets with DoubletFinder (McGinnis et al., 2019). Filtration and normalization was performed with Seurat (Hao et al., 2024; Satija et al., 2015) (nFeatureRNA 500–6,500; nCountRNA 500–25,000; %-mitochondrialRNA < 3%) and integrated (Korsunsky et al., 2019) to reduce batch effects. To ensure that downstream analyses were restricted to nuclei derived from LH neurons we further subsetted nuclei based on the expression of neuronal and LH marker genes (Mickelsen et al., 2019, 2017; Seifinejad et al., 2019) as well as absence for glial marker genes (see Methods). Our strategy yielded a total of 33,235 high-quality LH neuronal nuclei, 16,432 nuclei derived from female mice and 16,803 nuclei from males (Figure 1b). We calculated the odds ratio (OR) for nuclei counts between the sexes and found an OR of 1.0 (Figure 1c). Following the interpretation of ORs by Chen et al. (2010), all 33,235 nuclei were included for further analysis without down sampling.

### LH neurons exhibit sex-associated differences in clock and circadian signaling-associated gene expression

Our study focused on a manually curated list of genes encoding core circadian clock components, auxiliary feedback actuators as well as neuropeptide receptors for suprachiasmatic nucleus (SCN) neuron types to assess canonical circadian neuromodulatory pathways in female- and male-derived LH neurons. This included core clock genes (*Clock, Arntl, Npas2*), core repressors (*Per1, Per2, Per3, Cry1, Cry2*), transcriptional activators (*Nr1d1, Nr1d2, Rora, Rorb, Rorc)* and neuromodulator receptors for VIP (*Vipr1, Vipr2*), AVP (*Avpr1a, Avpr2*), gastrin-releasing peptide (GRP: *Grpr*), neuromedin-S (NMS: *Nmur1, Nmur2*), cholecystokinin, CCK: *Cckar, Cckbr*), as well as melatonin receptors (*Mtnr1a, Mtnr1b*) which also exhibit rhythmic expression over the 24-h period (Waly and Hallworth, 2015; Pinato et al., 2017). We collectively refer to these features as clock and circadian signaling-associated genes (Table 1), and used them as genes of interest (GOIs) for our analysis.

**Table 1:** Summary of clock and circadian signaling-associated gene symbols and names.

Gene symbol	Name
SCN-associated neuropeptide receptors	
*Vipr1*	Vasoactive intestinal peptide receptor 1
*Vipr2*	Vasoactive intestinal peptide receptor 2
*Avpr1a*	Arginine vasopressin receptor 1A
*Avpr1b*	Arginine vasopressin receptor 1B
*Avpr2*	Arginine vasopressin receptor 2
*Grpr*	Gastrin releasing peptide receptor
*Nmur1*	Neuromedin U/S receptor 1
*Nmur2*	Neuromedin U/S receptor 2
*Cckar*	Cholecystokinin receptor A
*Cckbr*	Cholecystokinin receptor B
Core clock genes	
*Clock*	Circadian locomotor output cycles kaput
*Arntl*	Brain and Muscle-ARNT-like (BMAL) 1
*Npas2*	Neuronal PAS domain protein 2
Core clock repressors	
*Per1*	Period 1
*Per2*	Period 2
*Per3*	Period 3
*Cry1*	Cryptochrome 1
*Cry2*	Cryptochrome 2
Auxiliary feedback components, transcriptional activators	
*Nr1d1*	Rev-Erb alpha
*Nr1d2*	Rev-Erb beta
*Rora*	ROR alpha
*Rorb*	ROR beta
*Rorc*	ROR gamma
Melatonin receptors	
*Mtnr1a*	Melatonin receptor 1A
*Mtnr1b*	Melatonin receptor 1B

Our first analysis examined the expression of clock and circadian signaling-associated genes across neuron types to evaluate gene expression in the LH globally. We observed expression of almost all GOIs in both female- and male-derived nuclei (Figure 1d), although at varied levels. Transcripts encoding *Avpr1b* were not detected in either sex. When ranked by effect size (log2 fold change, log2FC; Figure 1e) we found that several genes showed strong shifts in expression levels, including *Avpr1a* and *Mtnr1b* for female-derived nuclei, and *Vipr2* and *Avpr2* for male-derived nuclei.

Using a differential expression analysis (Wilcoxon rank-sum test) and the false discovery rate (FDR) to correct for multiple comparisons (Benjamini–Hochberg correction), we compared GOI expression between female- and male-derived LH neurons. We identified a total of 15 differentially expressed genes (FDR ^*^p-value < 0.05), 7 of which were enriched in female-derived LH nuclei (Figure 1f). This included genes encoding receptors *Avpr1a* and *Mtnr1b*, core repressors *Per2* and *Per3*, as well as transcriptional activators *Nr1d1, Nr1d2*, and *Rora*. In male-derived nuclei we found 8 differentially expressed genes (FDR ^*^p-value < 0.05), including *Vipr2* (Figure 1f). Other differentially expressed genes included those encoding SCN-associated neuropeptide receptors *Nmur2* and *Cckar*, the transcriptional activator *Rorb*, core repressors *Per1* and *Cry1* as well as core clock components *Clock* and *Npas2*. Taken together, these findings support the idea of sex-associated representations of circadian-linked neuromodulator receptor transcripts in LH neurons. It also indicates that female- and male-derived LH neurons may exhibit subtle changes in components of the intrinsic circadian oscillator and core feedback modulators at a relevant timepoint in the 24 h cycle: the onset of the dark period.

### Resolving LH neuron types

In our next set of analyses, our goal was to assess clock and circadian signaling-associated gene expression in defined LH neuron types, which have unique roles in controlling circadian-linked behaviors, like sleep and arousal (Bonnavion et al., 2016; Arrigoni et al., 2019). To do this, we merged the female and male datasets, ran a principal component analysis (PCA, Figure 6), performed dataset integration, and then unsupervised clustering with the Leiden algorithm. We visualized the resulting dataset using the Harmony-integrated PCA embeddings in a Uniform Manifold Approximation Projection (UMAP) plot. This analysis revealed 24 distinct clusters (Figure 2a) on the shared UMAP plot of both sexes (i.e., the female and male nuclei merged dataset). In the individual UMAP plots for female- (Figure 2b) and male-derived nuclei (Figure 2c) we found extensive overlap across all clusters. No clusters were sex specific, with each of the 24 clusters represented in the UMAP plots for female- (Figure 2d) and male-derived nuclei (Figure 2e). Taken together, these findings indicate that female- and male-derived LH nuclei exhibit similar cell type-organizations, consistent previous observations (Mickelsen et al., 2019), having all clusters represented in each sex and no evidence of sex-specific segregation.

**Figure 2:**
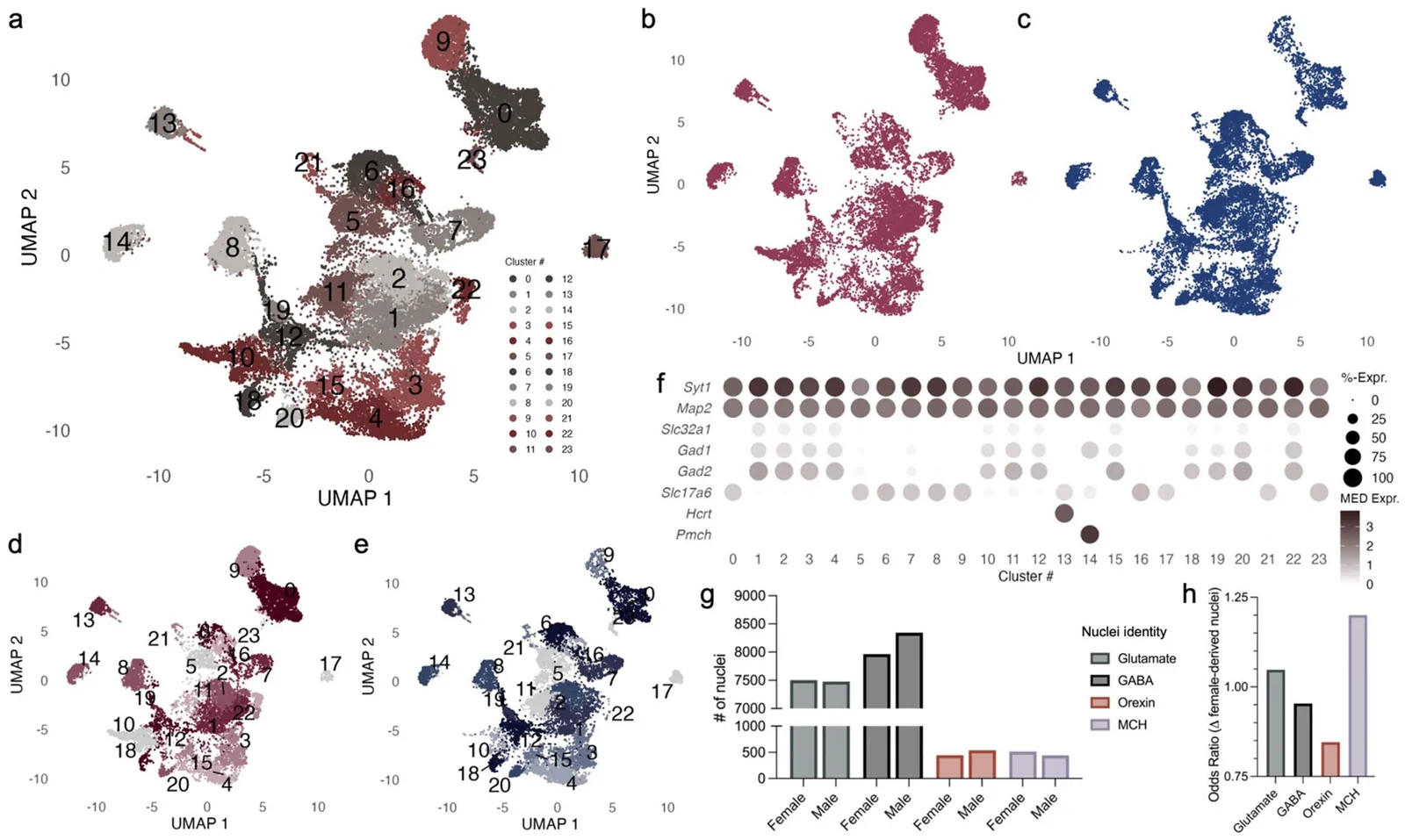
Identifying, classifying and subsetting LH neurons through cluster annotation. **(a)** Integrated Uniform Manifold and Projection (UMAP) plot depicting all LH nuclei (female and male) subsetted for analysis. Individual UMAP plots colored for female- **(b)** and male-derived LH nuclei **(c)** All 24 clusters were represented in nuclei derived of female **(d)** and male **(e)** mice. **(f)** Median normalized expression level (MED Expr.) and prevalence (percent expression, %-Expr.) of pan-neuronal marker genes (*Syt1, Map2*) and markers of LH neuron types across clusters. **(g)** Visualization of nuclei counts according to sex after annotation for classification. **(h)** Odds ratios comparing female- and male-derived nuclei counts according to neuron type classification.

To subset LH neuron types, we analyzed the expression of neurochemical marker genes for LH resident neuron types. This included markers for GABA (*Slc32a1, Gad1, Gad2*), glutamate (*Slc17a6*), orexin (*Hcrt*) and MCH (*Pmch*) neurons (Figure 2f), which we used to assign a neurochemical phenotype to clusters. We found 12 GABAergic clusters, 10 glutamatergic clusters, 1 orexinergic cluster and 1 MCH-associated cluster. Based on these annotations we resolved the relative abundance of LH neuron types in female- and male-derived samples. Although subpopulations of LH orexin and MCH neurons co-express glutamate (Chee et al., 2015) and GABA (Mickelsen et al., 2019, 2017), these populations were subsetted by neuropeptide expression and analyzed independently from the broader glutamatergic and GABAergic populations. In the female cohort, 7,964 nuclei were classified as GABAergic, 7,504 nuclei as glutamatergic, 448 nuclei as orexinergic, and 516 nuclei as MCH-associated (Figure 2g). Among male-derived nuclei, 8,343 nuclei were identified as GABAergic, 7,479 nuclei as glutamatergic, 539 nuclei as orexinergic and 442 as MCH-associated.

To determine if the resolved LH neuron types were proportionally represented between the sexes, we again calculated the OR. Following the same interpretation guidelines, this analysis revealed low ratios (Figure 2h) for glutamate- (OR = 1.05), GABA- (OR = 0.95), orexin- (OR = 0.85) and MCH-assigned nuclei (OR = 1.20). We then subsetted nuclei based on their assigned neurochemical phenotype, without down sampling, to compare clock and circadian signaling-associated gene expression in transcriptionally-resolved LH neuron types.

### Assessing clock and circadian signaling-associated gene motifs in female- and male-derived LH excitatory and inhibitory neurons

In our next study, we examined clock and circadian signaling-associated gene expression in the glutamatergic and GABAergic factions of female- and male-derived LH nuclei. Transcripts for almost every GOI was detected in both sexes and neuron types, except for *Avpr1b* which was absent across cohorts (Figures 3a, b). In the female-derived glutamatergic subpopulation, several gene features exhibited large effect sizes (Figure 3c), including *Avpr1a* and *Mtnr1b*. Following a differential gene expression analysis for GOIs (FDR ^*^p-value < 0.05), we found enrichment for transcripts encoding *Per2, Nr1d2, Rora* and *Avpr1a* (Figure 3d). In male-derived glutamatergic nuclei we found that *Vipr2* and *Avpr2* exhibited the greatest levels of change (Figure 3c). Differential expression analysis (FDR ^*^p-value < 0.05) further identified enrichment for transcripts encoding neuropeptide receptors *Vipr2, Cckar* and *Nmur2*, as well as core clock components *Npas2* and *Clock* (Figure 3d). These findings indicate that female-derived glutamatergic LH neurons may exhibit greater molecular representation of AVP signaling pathways, while male-derived excitatory neurons may be enhanced for VIP. The differences observed between the sexes for genes encoding components of the molecular circadian clock and neuromodulator signaling pathways may reflect coordinated differences in circadian-related transcriptional programs at ZT 12, and may be associated with biological sex, endocrine state, circadian phase, or an interaction among these variables.

**Figure 3:**
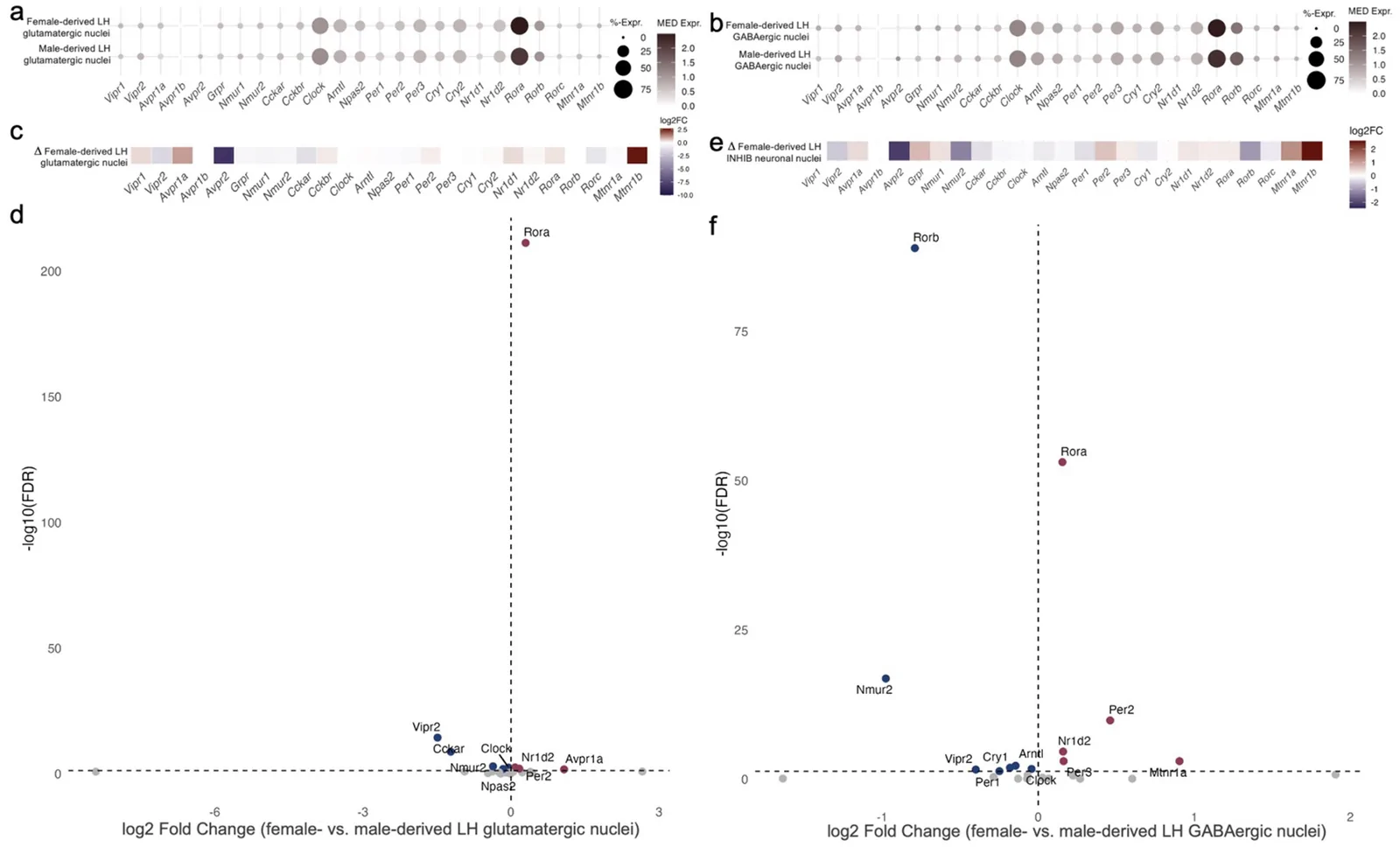
Clock and circadian signaling-associated gene expression in female- and male-derived LH glutamatergic and GABAergic neurons. Dot plot with median normalized expression levels of clock and circadian signaling-associated GOIs in both sexes for glutamatergic nuclei **(a)** and GABAergic nuclei **(b)**. **(c)** Heatmap depicting GOI expression as log2FC for effect sizes in glutamatergic nuclei, relative to female-derived nuclei. **(d)** Volcano plot of differentially expressed GOIs (Wilcoxon rank-sum test; FDR; Benjamini Hochberg correction, *p*-value < 0.05) for glutamatergic nuclei. **(e)** Heatmap depicting GOI expression as log2FC for effect sizes in GABAergic nuclei, relative to female-derived nuclei. **(f)** Volcano plot of differentially expressed GOIs showing enrichment for GABAergic nuclei (*p*-value < 0.05).

In the GABAergic subset of LH-derived nuclei, clock and circadian signaling-associated gene expression was similarly perturbed. In the female cohort, melatonin receptors (*Mtnr1a, Mtnr1b*) ranked highest for effect size (Figure 3e), whereas *Avpr2, Nmur2* and *Rorb* ranked highest in male-derived nuclei. *Nr1d2, Per2, Per3, Rora* and *Mtnr1a* were further identified as differentially expressed features (Figure 3f; FDR ^*^p-value < 0.05) showing enrichment in female-derived nuclei. Conversely, the male cohort was enriched for several genes including *Nmur2, Rorb, Vipr2, Per1, Cry1, Arntl* and *Clock*. Taken together, these findings suggest that glutamatergic and GABAergic LH neurons may exhibit sex-associated sensitivities to canonical circadian neuromodulatory pathways like AVP, VIP, CCK, NMS and melatonin through specific postsynaptic receptor types. We also identify emerging motifs for AVP and VIP signaling, with the enrichment of *Avpr1a* and *Vipr2* in female- and male-derived LH neurons (Figures 1f, 3d, 3f), respectively. Our results also indicate that the expression of genes encoding components of the core molecular clock and regulatory factors differ between female- and male- derived LH glutamatergic and GABAergic neurons at ZT12, and may be associated with biological sex, endocrine state, circadian phase, or an interaction among these variables.

### Clock and circadian signaling-associated gene expression in female- and male-derived LH peptidergic neurons

We next examined clock and circadian signaling-associated gene expression in LH orexin and MCH neurons. Transcripts for most features were detected across the sexes, except for *Avpr1b* and *Avpr2*, which were absent in both populations. Both sexes of *Hcrt*-expressing nuclei additionally lacked transcripts encoding *Mtnr1a* (Figure 4a), and all *Pmch*-expressing nuclei were also absent for *Vipr1* and *Avpr1a* (Figure 4b). Genes with the largest differences in expression levels included *Mtnr1b* for female-derived nuclei as well as *Vipr2* and *Rorc* in the male cohort (Figure 4c). Differential gene expression analysis identified a small subset of genes enriched both sexes (FDR ^*^p-value < 0.05), including *Npas2* enrichment in female-derived nuclei (Figure 4d), and *Nr1d2* in male-derived samples.

**Figure 4:**
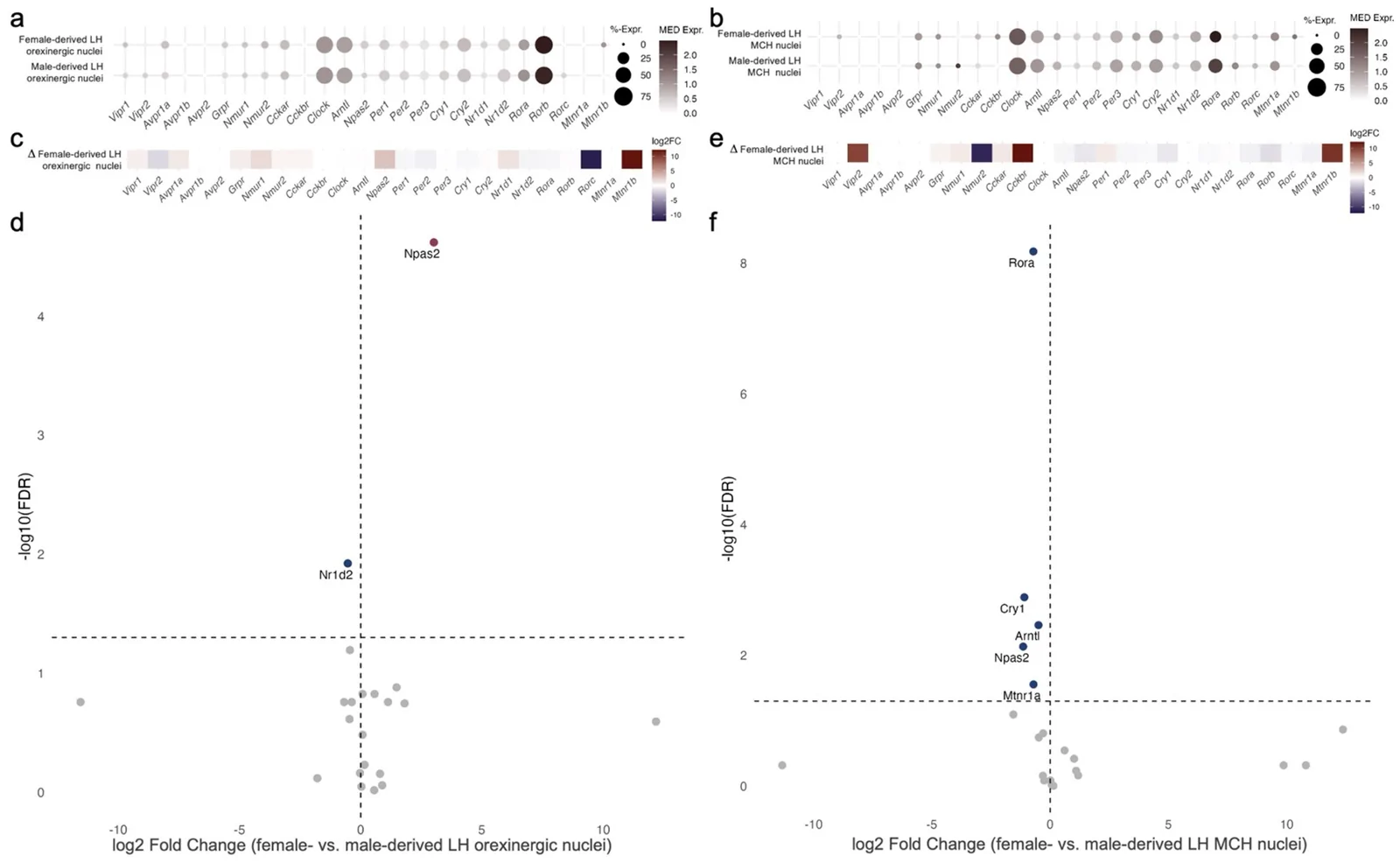
Clock and circadian signaling-associated gene expression in female- and male-derived LH orexin and MCH neurons. Dot plot with median normalized expression levels of clock and circadian signaling-associated GOIs in both sexes for orexinergic nuclei **(a)** and MCH-expressing nuclei **(b)**. **(c)** Heatmap depicting GOI expression as log2FC for effect sizes in orexinergic nuclei, relative to female-derived nuclei. **(d)** Volcano plot of differentially expressed GOIs (Wilcoxon rank-sum test; FDR; Benjamini Hochberg correction, *p-value < 0.05) for orexinergic nuclei. **(e)** Heatmap depicting GOI expression as log2FC for effect sizes for MCH-expressing nuclei, relative to female-derived nuclei. **(f)** Volcano plot of differentially expressed GOIs showing enrichment for MCH-expressing nuclei (*p*-value < 0.05).

When analyzing clock and circadian signaling-associated gene expression in MCH-expressing nuclei, several genes exhibited large effect sizes, including *Vipr2, Cckbr* and *Mtnr1b* (Figure 4e) in female-derived nuclei and *Nmur2* in the male cohort. Differential gene expression analysis (FDR ^*^p-value < 0.05) revealed that *Rora, Cry1, Arntl, Npas2*, and *Mntr1a* were enriched in male-derived MCH-associated nuclei (Figure 4f), with no differentially expressed genes identified in the female cohort. Collectively, our findings indicate that components of the core clock and auxiliary feedback modulators are differentially expressed between female- and male- derived LH peptidergic neurons at the onset of the dark phase.

### Expression of sex hormone receptors across LH neurons

In our final analysis, we examined sex hormone receptor prevalence in LH neurons by characterizing the expression of androgen receptor (AR, *Ar*), estrogen receptor 1 (ESR1, *Esr1*) and estrogen receptor 2 (ESR2, *Esr2*). We found in both sexes that all LH neurons expressed *Ar, Esr1* and *Esr2* with consistent patterns of expression (Figure 5a). Specifically, we found that transcripts encoding AR were expressed in the greatest abundance, followed by *Esr1* and *Esr2*, which exhibited the lowest expression levels in both female- and male-derived nuclei. Feature plots based on group UMAP embeddings (Figure 2a) visualize sex hormone receptor expression across nuclei for each LH neuron type (Figures 5b–e). Our findings show that sex steroid hormone receptors are expressed across LH neuron types, identifying candidate neuronal populations for androgen- and estrogen-mediated signaling.

**Figure 5:**
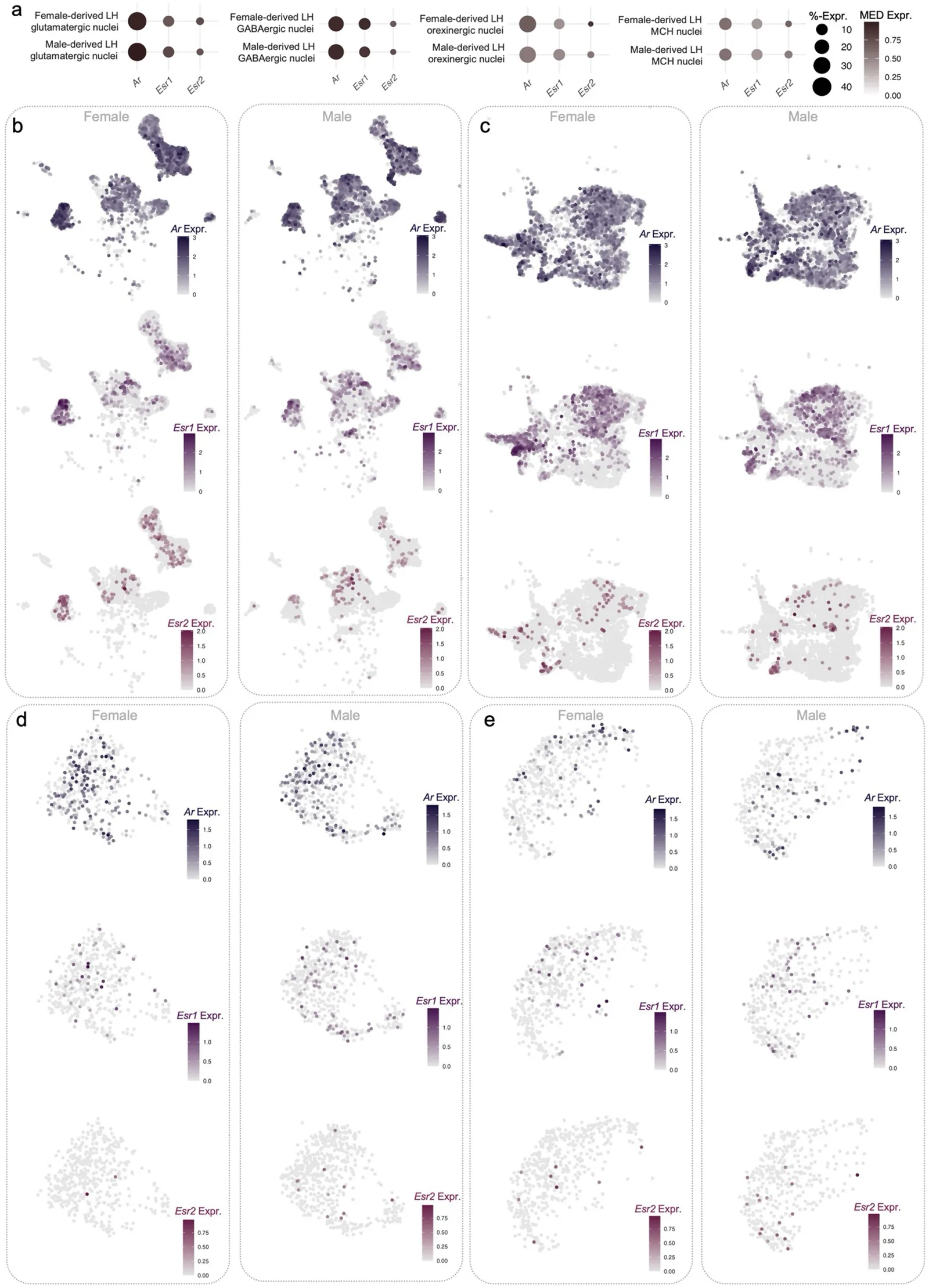
Expression of sex hormone receptors in female- and male-derived LH neurons. Sex hormone receptor expression was assessed in female- and male-derived nuclei. **(a)** Dot plots depict median normalized expression levels (MED Expr.) and abundance (%-Expr.) of transcripts in female- and male-derived LH neurons. Using the UMAP plots of all LH neurons for each sex (i.e., Figures 2d, e) the expression of *Ar, Esr1* and *Esr2* are visualized in glutamatergic **(b)**, GABAergic **(c)**, orexinergic **(d)** and MCH-expressing **(e)** nuclei.

## Discussion

The circadian system confers temporal organization to physiology and behavior, aligning cellular, molecular and systems-level processes with recurring environmental cycles. Although sex differences in circadian-linked physiological processes and behaviors oscillations have been described across species (Anderson and FitzGerald, 2020; Duffy et al., 2011; Santhi et al., 2016; Talamanca et al., 2023; Astafev et al., 2024), comparatively little is known about how molecular architectures associated with circadian-state regulation differ between the sexes. This question is particularly relevant outside of the SCN, where local oscillatory programs and circadian neuromodulatory signaling are thought to coordinate tissue- and circuit-specific physiological outputs across the broader circadian regulatory network. Here, we examined clock and circadian signaling-associated gene expression in female- and male-derived LH neurons, a region critically involved in the control of arousal, wakefulness, thermoregulation, feeding and behavioral state transitions (Bonnavion et al., 2016; Arrigoni et al., 2019). To our knowledge, this study provides the first comprehensive characterization of clock, select neuropeptide and sex hormone receptor gene expression using cell type resolved RNA sequencing within defined LH neurons. Our findings support a model in which sex-associated changes in gene expression within the LH emerges not through fundamentally distinct neuronal populations, but through distributed and coordinated differences in molecular signaling architectures.

Importantly, the overall cellular organization of the LH was highly conserved between sexes. Female- and male-derived nuclei exhibited extensive overlap across transcriptionally-resolved neuronal populations, with little evidence for sex-specific clustering or large differences in neuron type prevalence (Figures 2b–h). Instead, variation between female- and male-derived LH neurons at the onset of the dark phase emerged through moderate yet reproducible differences in the expression of clock-associated transcriptional regulators and circadian signaling-related receptors. Female-derived nuclei exhibited enrichment for genes associated with AVP signaling (*Avpr1a*), core clock repressors (*Per2, Per3*), and auxiliary transcriptional regulators (*Nr1d2, Rora)*. In contrast, male-derived nuclei exhibited enrichment for genes associated with VIP-, NMS- and CCK-related signaling (*Vipr2, Nmur2, Cckar*), core clock components (*Clock, Npas2, Arntl*), and transcriptional regulators (*Per1, Cry1, Rorb*). These motifs were reproducible across multiple LH neuron populations, suggesting coordinated sex-biased weighting of distinct circadian signaling pathways rather than large-scale divergence in transcriptional identity. Future experimentation delineating the sources of this variation according to biological sex, endocrine state, circadian phase, and the interaction among these variables will be formative to our understanding of diurnal processes.

Despite observations on sex dimorphism in chronobiology (Anderson and FitzGerald, 2020; Duffy et al., 2011) and chronophysiology (Santhi et al., 2016; Talamanca et al., 2023; Astafev et al., 2024) the influence of sex in within the SCN and throughout the broader circadian network are not fully understood. Sex hormones act on the SCN directly, where androgen and estrogen receptors exhibit graded expression by anatomical division and cell type. ARs are preferentially expressed in the SCN's ventral core (Jahan et al., 2025) and are associated with GRP and VIP neuronal subpopulations specifically. Interestingly, AR-expressing SCN neurons may be photo-responsive (Karatsoreos et al., 2007), inferring a potential mechanism where circulating steroid hormones may directly influence circadian timing, consistent with testosterone altering the magnitude of light-induced phase shifts in a dose-dependent manner (Butler et al., 2012). Although ESR1 is sparsely expressed in the SCN (Jahan et al., 2025), ESR2 is abundantly expressed and spatially organized along the rostrocaudal axis throughout the shell and core (Vida et al., 2008). Recent evidence suggests that estrogen signaling via ESR2 in SCN neurons and astrocytes stabilizes firing synchrony (Schlaeger et al., 2024), identifying a previously unrecognized mechanism through which sex hormones may influence circadian network organization.

Beyond the SCN and within the distributed circadian network, the LH is emerging as an active component for the integration of temporal information like the DMH. LH orexin and MCH neurons exhibit robust diurnal rhythms in activity (Hassani et al., 2009; Mileykovskiy et al., 2005) to control circadian-linked behaviors like feeding, arousal and sleep (Bonnavion et al., 2016; Arrigoni et al., 2019). Orexin neurons specifically are a critical actuator of circadian output, as they regulate circadian-linked arousal via direct projections from the DMH and indirectly from the SCN (Chou et al., 2003). Recent work demonstrated that orexin neurons project to the SCN and modulate the circadian period (Hung et al., 2025), indicating that LH-derived neural signaling contributes to principal pacemaker regulation directly. Consistent with this emerging idea, our findings characterize the abundance of clock and circadian signaling-associated pathways in LH neurons at the onset of the dark phase. While the LH exhibits rhythmic clock gene expression (Abe et al., 2002), it is still unclear if it possesses endogenous rhythmicity comparable to that of the SCN or DMH. Although our single-time point analysis cannot determine if the observed transcriptional changes reflect endogenous oscillatory properties of the LH, it instead nominates molecular substrates through which LH neurons may participate in the distributed circadian network or differentially weight molecular clock components. Taking together the functional, anatomical and transcriptional properties of LH neurons infers a role for this region in integrating circadian timing signals regarding behavioral and physiological state.

Given the single time point design of this study, the sex-associated measures of transcript abundance do not permit conclusions regarding rhythmic gene expression, phase relationships or oscillatory amplitude across the 24-h cycle. Instead, this study provides an assessment of factors associated with the molecular clock at a biologically-relevant timepoint to provide context regarding transcript abundance in female- and male-derived LH neurons at the onset of the dark phase. As such, we interpret our findings with caution and advocate for future experimentation assessing humoral cues, reproductive cycle timing at multiple circadian timepoints within this framework.

One of the more notable observations was the enrichment of transcripts encoding CLOCK (*Clock*), BMAL1 (*Arntl*) and NPAS2 (*Npas2*) in male-derived LH neurons across several neuronal populations (Figures 1f, 3d, 4f). CLOCK and BMAL1 form the principal transcriptional activator complex of the mammalian circadian oscillator, driving rhythmic expression of downstream clock-controlled genes and stabilizing circadian phase relationships (Abe et al., 2022; Bunger et al., 2000). NPAS2 functions as a partially redundant CLOCK paralog that contributes to circadian amplitude and temporal precision (DeBruyne et al., 1972). In parallel with these observations, female-derived LH neurons exhibited enrichment for *Per2* and *Per3*, whereas *Per1* and *Cry1* were preferentially enriched in male-derived nuclei. PER and CRY proteins form inhibitory complexes that suppress CLOCK:BMAL1-mediated transcription through the negative limb of the circadian feedback loop (Sato et al., 2006; Liu et al., 2007) via transcriptional repression of the CLOCK:BMAL1 complex, also present at elevated levels. Female-derived nuclei also exhibited enrichment for transcripts encoding REV-ERBα (*Nr1d1*), REV-ERBβ (*Nr1d2*), and RORα (*Rora*), components of auxiliary stabilizing loops that regulate rhythmic *Bmal1* expression and contribute to circadian amplitude and phase maintenance (Sato et al., 2004; Abe et al., 2022; Cho et al., 2012). Importantly, these findings should not be interpreted as evidence for distinct circadian oscillators, phase relationships, oscillatory amplitudes, stabilization, or phase-dependent control between the sexes. We favor the interpretation that LH neuron populations may engage circadian transcriptional machinery through different molecular weighting strategies at the onset of the active phase, tuned to their innate rhythm. The consistent enrichment of specific repressor paralogs and auxiliary stabilizing components may indicate differential representation of these transcriptional pathways in female- and male-derived LH neurons, although further experimentation is required to specify contributions.

In addition to intrinsic clock-associated genes, in female- and male-derived LH neurons we observed motifs in the expression of receptors associated with circadian neuromodulatory pathways. Across LH neurons, and particularly within glutamatergic populations, female-derived nuclei exhibited enrichment for transcripts encoding AVP receptor 1A (*Avpr1a*, Figures 1f, 3d), whereas male-derived nuclei exhibited elevated expression of AVP receptor 2 (*Avpr2*, Figures 1e, 3c, 3e). AVP signaling is a principal component of SCN output circuitry and contributes critically to circadian synchrony and phase regulation (Mieda et al., 2015). Although AVP expression within the SCN itself does not appear strongly sexually dimorphic (Krajnak et al., 1998). vasopressin receptors exhibit sex-dependent organization in the CNS (Dubois-Dauphin et al., 1996). Our findings suggest that neuropeptide signaling within the LH may arise, at least in part, through distinct postsynaptic mechanisms for behavioral and physiological processes associated with the female sex.

Similarly, male-derived LH neurons exhibited enrichment for *Vipr2, Nmur2* and *Cckar* (Figures 1e, 1f, 3c–f) suggesting enhanced representation of signaling pathways associated with VIP, neuromedin and CCK-related circadian modulation. VIP signaling is essential for synchrony and amplitude maintenance within SCN networks and contributes to downstream physiological rhythmicity (Todd et al., 2020; Aton et al., 2005). NMS is proposed to regulate circadian rhythmicity through pacemaker function and coupling activity between oscillator cells circadian network synchrony (Lee et al., 2015), whereas CCK-associated pathways may participate in circadian adaptation and entrainment under altered photoperiod conditions (Xie et al., 2023). Although the precise sources of these neuropeptides to the LH remain unresolved, and cannot be inferred directly from the present dataset, the coordinated enrichment of these receptor pathways infers that LH neurons derived of either sex may differentially weight neuromodulatory inputs associated with the SCN.

Sex steroid signaling is recognized as an important regulator of hypothalamic physiology and behavior (Calvigioni et al., 2023), but their molecular representation across LH neurons was incompletely characterized. We observed the expression of three sex steroid receptors across all LH neuron types (Figure 5a), where notably, receptor transcript abundance was consistently highest for *Ar*, moderate for *Esr1* and lowest for *Esr2*. The expression of ESR1 in LH neurons is of particular importance given its function as a ligand-activated transcription factor driving the expression of *Per2* (Gery et al., 2007) and *Clock* (Xiao et al., 2014) in hypothalamic neurons. These findings provide a molecular framework through which androgenic and estrogenic signaling may contribute to dimorphic hypothalamic function in the sexes, including the process of gene expression.

Sex dimorphism in gene expression are documented across numerous brain regions, where sex-associated transcriptional programs are hypothesized to contribute to region and cell type-specific functions. The level of transcriptional remodeling observed between the sexes is dynamic, with reports of transcriptionally-distinct subpopulations between the sexes (Kim et al., 2019) as well as differential weighting of gene expression according to neuron type (Chen et al., 2019). However, the source of these observations is yet unknown. Gonadectomy in female and male mice with sex hormone replacement infers a restricted role for sex hormone signaling in sex-based transcriptional remodeling (Xu et al., 2012). Rather, sex hormone signaling may affect the expression of targeted GOIs where, interestingly, *Cckar* is specifically recognized for its sex hormone-induced dimorphic expression (Welch et al., 2019). Reports on the magnitude of transcriptomic remodeling across the estrous cycle is variable according to brain region (Chen et al., 2025; DiCarlo et al., 2017) and in general highly understudied. Of note, one report in the hypothalamus found that only ~1.3% of detectable transcripts exhibited altered expression levels across all four stages of the murine estrous cycle. Our experiment design on gonad-intact animals without estrous staging precludes observations on the influence of sex hormones and reproductive cycles, but represents an initial effort to characterize sex-differences in the LH. A comprehensive understanding of sex-specific transcriptional regulation will require an experimental design that addresses all of these modalities to distinguish the effects of biological sex and other sex-associated variables.

Several other limitations should be considered when interpreting the present findings. First, tissue was collected at a single circadian timepoint (ZT12), corresponding to dark-phase onset in nocturnal animals. Consequently, our analyses cannot resolve oscillatory amplitude, phase relationships, or dynamic transcriptional trajectories across the circadian cycle. The observed sex-associated effects therefore likely reflect an active-phase transcriptional configuration rather than stable circadian properties across the full 24-h period. Future circadian time-course studies will be necessary to determine how these molecular signatures evolve across behavioral state transitions and whether sex-associated transcriptional weighting varies according to circadian phase.

Taken together, our findings support a model in which the molecular logic of clock and circadian signaling-associated targets difsfers subtly but reproducibly between sexes within shared LH neuronal architectures. Female-derived LH neurons exhibited enrichment for specific AVP signaling pathways and auxiliary clock regulators whereas male-derived nuclei exhibited enrichment for core positive-limb oscillator components and receptors associated with VIP-, NMS- and CCK-linked signaling. Indeed, the absence of sex-specific neuronal clusters suggests that molecular dimorphism in the LH may emerge primarily through differential tuning of shared neuronal populations rather than through sex-restricted cell classes. Rather than indicating fundamentally distinct neuronal populations, LH neuron populations may engage circadian transcriptional machinery through different molecular weighting strategies, tuned to their innate rhythm. The consistent enrichment of transcriptional activators, repressor paralogs, auxiliary stabilizing components and neuropeptide receptors may indicate differential representation of these transcriptional pathways in female- and male-derived LH neurons. Accordingly, we interpret these findings as sex-associated transcriptional signatures observed at a defined physiological state rather than stable molecular properties of female and male LH neurons.

## Methods

### Animals

Adult female (*n* = 4) and male (*n* = 4) *Vgat-Ires*-cre mice (12 weeks old) were housed under a 12:12 light-dark cycle with food and water provided ad libitum. All procedures were performed at the University of California Davis, approved by the Institutional Animal Care and Use Committee and in accordance with guidelines set by the National Institutes of Health.

### Viral injections

The animals used in this study received stereotaxic injections of a cre-dependent adeno-associated virus (Tervo et al., 2016) (AAV, AAV2retro-CAG-FLEX-rc[Jaws-KGC-GFP-ER2]) (Chuong et al., 2014) for retrograde labeling of neurons in an independent experiment. The viral construct (Addgene CAT# 84445) was delivered to the ventral tegmental area (VTA) to retrogradely label VGAT neurons that project to this region. Tissue was collected 6 weeks after viral infusion surgeries. The use of AAV2retro vectors in parallel with single-cell RNA sequencing experiments has been validated and reported to have minimal effects on transcriptional programs beyond transgene expression (Xu et al., 2024). AAVs are considered minimally perturbative in the absence of actuator activation (i.e., JAWS) and is unlikely to explain the observed differences in gene expression between the sexes.

### Tissue collection

For tissue collection, mice were removed from the colony room prior to dark phase onset (ZT 11:45). Mice were deeply anesthetized by isoflurane (3% in oxygen, 2L/min) and decapitated. The brain was removed from the skull and submerged in ice cold calcium- and magnesium-free phosphate buffer saline (PBS) for 3 min. Using a 1.0mm brain matrix, the brain was coronally sectioned between −1.0mm and −2.0mm anterior-posterior (AP) from bregma to acquire a tissue section containing the LH. Using a 1.0mm tissue biopsy punch, the LH was bilaterally microdissected and frozen on dry ice. Animals were sacrificed over a 40 min period (ZT 12–12:40) in alternating order by sex to control for potential confounding effects for time and light exposure. Tissue was stored in liquid nitrogen until processing.

### Nuclei isolation

Frozen LH tissue was thawed and aggregated by sex for a total of 8 LH pellets per sex. LH pellets derived from female or male animals were processed separately. Nuclei isolation was performed using the Chromium Nuclei Isolation kit with RNase inhibitor from 10X Genomics (v2, PN-1001101) following the manufacturers guidelines. A 5 min incubation in lysis buffer was sufficient to adequately release nuclei from cells (cell viability, female-derived nuclei = 1.3%, male-derived nuclei = 0.6%).

### Single nucleus RNA sequencing

Quality control was performed by 10X Genomics staff using a Luna Dual Fluorescence Cell counter to assess cell viability and count nuclei. The 10X Genomics Chromium GEM-X Single Cell3′ v4 Gene Expression kit was used to generate single-nucleus barcoded emulsions with beads, targeting ~20,000 nuclei per sample. Nuclei suspensions from either sex were loaded into 2 wells on the same chip, generating 2 samples per sex (4 samples total). Barcoded libraries were constructed according to the manufacturers protocol. After complimentary DNA (cDNA) generation, cDNA was amplified for 13 PCR cycles and libraries were prepared using the AVITI platform (Element Biosciences, United States). Library complexity metrics prior to quality control and filtration, including cell recovery and sequencing depth are summarized in Table 2.

**Table 2:** Library complexities metrics prior to quality control and filtration.

Sample	No. of nuclei recovered	Mean reads/nuclei	Median genes/nuclei	Median UMI/nuclei
Female 1	23,916	19,805	2,468	5,168
Female 2	23,993	21,164	2,530	5,369
Male 1	18,602	26,491	3,206	7,244
Male 2	18,589	28,317	3,284	7,530

### Data processing, filtration and LH enrichment

FASTQ files were processed using the 10X Genomics CellRanger pipeline (version 8.0.1) and a transcriptome reference based on the GRCm38 Genome Assembly (mm10). Sparse count matrix files (.h5) produced by CellRanger count were imported into R Studio for processing with Seurat (Satija et al., 2015) v5 (Hao et al., 2024). On each dataset, ambient RNA was removed using SoupX (Young and Behjati, 2020) and the estimated global contamination fraction (rho max = 0.05). Doublet removal was performed with DoubletFinder (McGinnis et al., 2019) and an estimated doublet fraction of 7.5%. Using standard Seurat settings, data filtration was performed for nFeature RNA (>500, < 6,500), nCount RNA (< 30,000) and percent mitochondrial RNA (< 3%). Datasets were merged according to sex, log-normalized and scaled using the 2,000 most variable features. Principal component analysis (PCA) and unsupervised clustering with the Leiden algorithm were performed using an appropriate number of PCs (range nPCs = 12–25; Figure 6) and resolution (res = 0.5) for each dataset to annotate cell types. Cluster defining genes were assessed using the FindAllMarkers function (log2 fold change > 0.25, minimum percent: 25%). Clusters with mitochondrial genes highly represented in the top 5 cluster-defining genes, or ambiguous cluster markers were removed. To minimize contamination from adjacent brain regions, clusters enriched for markers characteristic of non-LH regions were also removed. The remaining nuclei were iteratively reprocessed (scaling, identification of variable features, PCA, unsupervised clustering, identification of cluster defining genes) and used for subsequent analyses.

**Figure 6:**
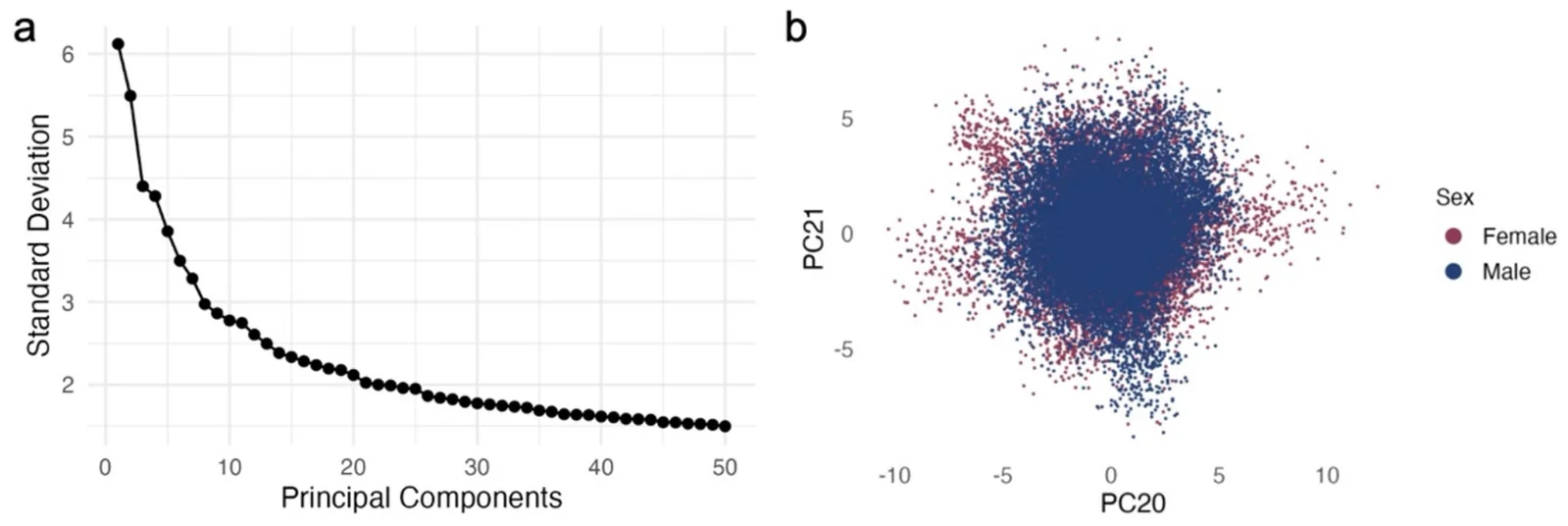
PCA of LH neuronal nuclei. **(a)** Elbow plots depicting standard deviation for each PC (*n*= 1-50PCs) in female- and male-derived LH nuclei. **(b)** PCA for the selected number of PCs (*n*= 21).

### Subsetting neuron-derived nuclei

Using canonical cell type markers (Table 3) we identified 10 resident CNS cell types. Clusters enriched in non-neuronal genes were removed to subset neuron-derived LH nuclei. After this, datasets were merged and metadata was modified to include experimental details (i.e., sex, sequencing run). Harmony (Korsunsky et al., 2019) was used to correct for batch effects and technical artifacts associated with tissue processing, sequencing and library preparation. Library complexity was re-assessed to ensure sequencing depth and gene detection was comparable between groups prior to analysis, summarized in Table 4. After this scaling with variable features, PCA (nPCs = 21) and clustering (res = 0.5) were performed again on the purified neuronal dataset and is presented in Figure 2. UMAP plots were generated based on the Harmony-integrated PCA embeddings and utilized to annotate, identify and subset LH neuronal populations for subsequent analyses.

**Table 3:** Cell type marker genes.

Cell type, region	Gene symbol	Gene name
Neuron	*Snap25*	Synaptosomal-Associated Protein 25
	*Rbfox3*	RNA Binding Fox-1 Homolog 3 (NeuN)
	*Tubb3*	Tubulin Beta 3 Class III
	*Map2*	Microtubule-Associated Protein 2
	*Syt1*	Synaptotagmin 1
LH	*Dlx1*	Distal-Less Homeobox 1
	*Dlx2*	Distal-Less Homeobox 2
	*Fox1b*	Forkhead Box B1
	*Lhx1*	LIM Homeobox 1
	*Lhx9*	LIM Homeobox 9
	*Otp*	Orthopedia Homeobox
Oligodendrocyte	*Mobp*	Myelin-Associated Oligodendrocyte Basic Protein
Astrocyte	*Slc4a4*	Solute Carrier Family 4 Member 4
Microglia	*Vcan*	Versican
Oligodendrocyte precursor cells	*Ctss*	Cathepsin S
Mural cells	*Pdgfrb*	Platelet-Derived Growth Factor Receptor Beta
Ependymal cells	*Tmem212*	Transmembrane Protein 212
Endothelial cells	*Flt1*	Fms-Related Tyrosine Kinase 1
Perivascular macrophages	*Mr1*	Mannose Receptor C-Type 1

**Table 4:** Library complexities metrics after quality control, filtration, and subsetting neuron-derived nuclei.

Sample	No. of nuclei	Mean genes/nuclei	Median genes/nuclei	Median UMI/nuclei
Female	16,432	3,652	3,632	7,951
Male	16,803	3,627	3,614	7,863

### Targeted gene expression and statistical analyses

Although we performed whole-transcriptome gene expression profiling, a predefined panel of canonical clock and circadian signaling-associated genes (genes of interest, GOIs) was curated to examine transcriptional differences between female- and male-derived nuclei (Table 1). Normalized expression values were extracted from the RNA assay (data layer) of the integrated Seurat Object where nuclei were annotated according to sex. To assess GOI abundance, the percentage of expressing nuclei was calculated as the proportion of nuclei with normalized expression values greater than zero relative to the total number of nuclei in that group. We also calculated median normalized expression values for each GOI. Data is presented as dot plots or feature plots based on UMAP embeddings from the harmonized object. To assess GOI differential expression, mean expression levels were calculated for female- and male-derived nuclei. Log2 fold change (log2FC) for each GOI using a small pseudocount (1e-6) to avoid division by 0. Statistical significance was assessed using the Wilcoxon rank-sum test and *p*-values were adjusted for multiple comparisons using the Benjamini–Hochberg correction for the false discovery rate (FDR). Genes with an FDR < 0.05 were considered significantly different between the sexes. Consistent with best practices in cell type-resolved gene expression profiling (Luecken and Theis, 2019), GOIs were also discussed based on effect size (log2FC) to avoid over-reliance on *p*-values and identify features with strong sex-biases. Because our objective was a hypothesis-driven evaluation of a predefined set of circadian and circadian-signaling associated genes, statistical testing was restricted to this a priori gene panel rather than performing a transcriptome-wide analysis DEGA.

## Data Availability

The data supporting the findings of this study are available from the corresponding authors upon reasonable request.
